# Proton-Translocating NADH–Ubiquinone Oxidoreductase: Interaction with Artificial Electron Acceptors, Inhibitors, and Potential Medicines

**DOI:** 10.3390/ijms252413421

**Published:** 2024-12-14

**Authors:** Vera G. Grivennikova, Grigory V. Gladyshev, Tatyana V. Zharova, Vitaliy B. Borisov

**Affiliations:** 1 Department of Biochemistry, Faculty of Biology, Lomonosov Moscow State University, 119234 Moscow, Russia; vgrivennikova@mail.ru (V.G.G.); gg.nurfed@gmail.com (G.V.G.); tzharova2018@gmail.com (T.V.Z.); 2 Belozersky Institute of Physico-Chemical Biology, Lomonosov Moscow State University, Leninskie Gory, 119991 Moscow, Russia; 3 Faculty of Bioengineering and Bioinformatics, Lomonosov Moscow State University, Leninskie Gory, 119991 Moscow, Russia

**Keywords:** membrane protein, molecular bioenergetics, respiratory chain, NADH–ubiquinone oxidoreductase, electron transfer, proton translocation, A/D transition of complex I

## Abstract

Proton-translocating NADH–ubiquinone oxidoreductase (complex I) catalyzes the oxidation of NADH by ubiquinone accompanied by the transmembrane transfer of four protons, thus contributing to the formation of a proton motive force (*pmf*) across the coupling membranes of mitochondria and bacteria, which drives ATP synthesis in oxidative phosphorylation. In recent years, great progress has been achieved in resolving complex I structure by means of X-ray crystallography and high-resolution cryo-electron microscopy, which has led to the formulation of detailed hypotheses concerning the molecular mechanism of coupling of the redox reaction to vectorial proton translocation. To test and probe proposed mechanisms, a comprehensive study of complex I using other methods including molecular dynamics and a variety of biochemical studies such as kinetic and inhibitory analysis is required. Due to complex I being a major electron entry point for oxidative metabolism, various mutations of the enzyme lead to the development of severe pathologies and/or are associated with human metabolic disorders and have been well documented. This review examines current information on the structure and subunit composition of complex I of eukaryotes and prokaryotes, reactions catalyzed by this enzyme, and ways to regulate them. The review also discusses biomedical aspects related to the enzyme in light of recent findings.

## 1. Introduction

Proton-translocating NADH–ubiquinone oxidoreductases of the mitochondrial respiratory chain (complex I) and prokaryotic plasma membranes (NDH-1) are the primary enzymes that catalyze the oxidation of NADH by ubiquinone. This reaction is coupled with the vectorial transmembrane transfer of four H^+^ ions and the consequent energy conservation in the form of an electrochemical gradient across the coupling membrane (proton motive force, *pmf*). The energy conserved is used primarily for ATP synthesis in oxidative phosphorylation as well as for ion transport and shift of other chemical equilibria [1]:

$$\mathrm{NADH}+Q+H^{+}+4{\vec{H}}_{\mathrm{in}}^{+}↔\;{\mathrm{NAD}}^{+}+{\mathrm{QH}}_{2}+4{\vec{H}}_{\mathrm{out}}^{+}$$

This reaction provides the main pathway for oxidation of NADH formed during the oxidative decomposition of carbohydrates, fatty acids, and proteins and, thus, maintains the physiological NAD^+^/NADH ratio necessary for an efficient turnover of central catabolic pathways, such as glycolysis, tricarboxylic acid cycle, and β-oxidation of fatty acids. Ubiquinol, produced by complex I, passes those reducing equivalents down the respiratory chain to the terminal acceptor—oxygen, and this is coupled to the transmembrane transfer of six more protons. Thus, complex I not only contributes approximately 40% to the total energy storage during the transfer of electrons from NADH to molecular oxygen but also provides electrons to other proton-translocating enzymes of the mitochondrial respiratory chain or the prokaryotic plasma membrane: cytochrome *b-c*_1_ complex and cytochrome *c* oxidase.

Complex I is the main source of reactive oxygen species (ROS) in the respiratory chain [2]. The imbalance between the rate of ROS formation and the potential ability of the cell antioxidant defense to deactivate them leads to irreversible destruction of the genetic apparatus of cells, oxidation of proteins and lipids, and other pathophysiological consequences. Due to the central role of complex I in energy metabolism, various mutations in the enzyme molecule lead to the development of severe pathologies and are also associated with various metabolic disorders [3].

In recent years, using the methods of X-ray crystallography and high-resolution cryo-electron microscopy (cryo-EM), a great breakthrough has been achieved in resolving complex I structures from various sources: *Thermus thermophilus* [4], *Escherichia coli* [5], and *Paracoccus denitrificans* [6] (bacteria); *Yarrowia lipolytica* [7] and *Chaetomium thermophilum* [8] (fungi); *Drosophila melanogaster* (insects) [9]; *Bos taurus* [10,11], *Ovies aries* [12,13], *Mus musculus* [14,15], and *Sus scrofa* [16] (mammals). The results obtained are of great importance and bring us closer to elucidating the mechanism of coupling between the redox reaction catalyzed by complex I and the vectorial translocation of protons. This review examines recent knowledge on subunit composition and structure of complex I in the respiratory chains of prokaryotes and eukaryotes; the structure of the substrate-binding centers of the enzyme, its interaction with artificial electron acceptors and specific inhibitors, as well as possible ways of complex I activity regulation.

## 2. The Structure of Complex I

### 2.1. Subunit Composition of Complex I

The structure of complex I, one of the most complicated components of the respiratory chain, became available from the pioneering studies of Sazanov’s group, when the complete atomic structure of an enzyme from the bacterium *T. thermophilus* was resolved using X-ray crystallography. Bacterial complex I includes a minimal set of 13–14 so-called core subunits that catalyze NADH–quinone oxidoreductase reaction. The total molecular mass of the enzyme is about 550 kDa [17,18]. The genes encoding the core subunits of complex I are usually combined into a single gene cluster or operon [19]. Complex I has an L-shaped structure and consists of two domains. The peripheral domain (PD), consisting of 7 subunits, protrudes into the cytoplasm of the bacterial cell, while another 7 subunits of the membrane domain (MD) are localized in the cytoplasmic membrane. Complex I includes several redox components: a flavin mononucleotide (FMN) and 8–9 iron–sulfur (Fe-S) clusters essential for electron transfer from NADH to a natural quinone electron acceptor (bacteria employ ubiquinone or menaquinone, as in the case of thermophilic bacterium *T. thermophilus*) [17,20].

In eukaryotes, complex I is much more complicated than in bacteria. For example, NADH–ubiquinone oxidoreductase of bovine heart mitochondria consists of 44 different subunits with a total molecular mass of about 1 MDa [21], among those, the SDAP subunit (mitochondrial acyl-transferring protein) is present in two copies [10]. Nevertheless, the enzyme contains the same minimum set of 14 subunits that are present in bacterial complex I [1]. Since the literature uses a different nomenclature of core subunits, for a more convenient comparison, Table 1 shows the designations of the homologous subunits of complex I from various species.

**Figure 1:**
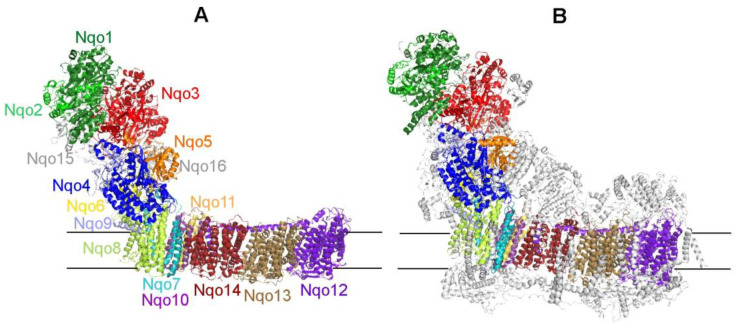
The structure of bacterial (**A**) and mammalian (**B**) complex I. The core subunits of *T. thermophilus* (**A**) are colored in their individual color. Homologous subunits of *O. aries* enzyme (**B**) are highlighted in the same way. Supernumerary subunits are colored in gray. The lines indicate the approximate location of the membrane.

Figure 1 shows the structures of complex I of *T. thermophilus* (PDB 4HEA [4]) and *Ovis aries* (PDB 5LNK [12]). A spectacular similarity in the shape and relative location of homologous subunits is noted. In complex I structure, several modules can be designated, according to subunits’ localization and functions [23] (Table 1). The Nqo1 subunit containing the nucleotide-binding center is located in the upper part of the PD of the *T. thermophilus* enzyme. Together with the Nqo2 and Nqo3 subunits, it forms a three-subunit N-module, whose function is the oxidation of NADH (Table 1). Proton-pumping P-module of the MD combines Nqo subunits 7, 8, and 10–14; three of these (Nqo 12–14) are highly homologous to bacterial Mrp cation/H^+^ antiporters [24,25] and, therefore, were suggested as direct participants in the vectorial proton transfer coupled with NADH oxidation [23,26]. The connecting Q-module, which is a part of the PD, contains Nqo subunits 4–6 and 9 and is located on the interface between PD and MD. This module contains the ubiquinone binding center formed by PD subunits Nqo 4 and 6, as well as MD subunits Nqo 7 and 8 [4].

In mammalian complex I, some of the supernumerary subunits have tissue-specific isoforms. Thus, the NDUFV3 subunit (*human* nomenclature) in mature cells is represented by a short 10 kDa isoform, whereas in the culture of growing cells and in the immature enzyme, a long 50 kDa isoform is found. The exception to this is the brain tissue, where mature enzyme contains a long 50 kDa isoform [27].

The function of 31 supernumerary subunits of the eukaryotic enzyme remains a mystery [28]. It is currently unknown why such a complicated structure arose as a result of natural selection, while all the main functions of complex I are quite successfully realized by simply arranged bacterial enzymes that catalyze the NADH–ubiquinone oxidoreductase reaction with the same energy yield as eukaryotic complex I [29,30]. It can only be assumed that additional subunits either participate in the correct assembly of the enzyme, or the presence of such subunits is associated with a possible precision regulation of complex I activity, or complex I, due to the presence of additional polypeptides, has hidden, yet unknown activities [7,31,32,33,34].

Supernumerary subunits are also found in bacterial enzymes. Thus, the complex I of *T. thermophilus* and related bacteria includes a frataxin-like subunit Nqo15, homologous to the mitochondrial protein that regulates iron ion homeostasis and participates in the biogenesis of Fe-S centers [18]. This subunit is encoded outside the *nqo* operon, which includes the genes of 14 core subunits of complex I [18]. In addition, another hydrophobic subunit Nqo16 was found in complex I, which is not important for the redox reaction and proton-translocating activity but is necessary for the proper assembly of protein crystals. The complex I of the soil bacterium *P. denitrificans* contains three additional subunits that are homologous to the supernumerary subunits of the mitochondrial enzyme: B17.2, AQDQ/18, and 13-kDa (*bovine* nomenclature) [35]. As in the case of the *T. thermophilus* enzyme, supernumerary subunits of the *P. denitrificans* complex I were encoded by different regions of chromosome 1 located outside the *nqo* operon. It should be noted that among all the studied microorganisms, the complex I from *P. denitrificans* has the highest homology with the human enzyme. In the PD domain, the similarity of subunits reaches 60–70%, whereas for *T. thermophilus* and *E. coli* enzymes, only 30–40% [35]. This confirms the hypothesis about the origin of modern mitochondria as a result of the symbiosis of α-proteobacteria and eukaryotic cells. Bacterial complex I of symbionts has complicated structures in the course of evolution, including many additional subunits [36].

In the cryo-EM structures of complex I, many (up to 3000) water molecules are detected, mainly located in the PD and in the external part of the MD, but also located in the inner part of the enzyme [5,11,13,15,16,37,38]. The role of the “internal” water associated with protein structures is significant; it participates in providing contacts between amino acid residues of protein and in binding substrates in the active sites through the formation of water-bridged hydrogen bonds. Water molecule displacement leads to rearrangements in the enzyme structure, which are important for catalysis. In addition, hydration is a key point for the transport function of proteins, as well as for the formation of Grotthus-competent pathways for proton transfer.

### 2.2. The Nucleotide-Binding Center of Complex I

The initial information about the structure of the nucleotide-binding center was obtained in Sazonov’s laboratory from the study of complex I of the thermophilic bacterium *T. thermophilus* [20,39]. Recently, the basic principles of the active center structure were confirmed by X-ray crystallography by the example of another thermophilic bacterium *A. aeolicus* [40,41,42]. The higher resolution of the structure reveals the presence of water bridges that participate in substrate binding and also provide contacts between amino acid residues lining the surface of the active center [40,42]. The nucleotide-binding site of complex I is a deep solvent-accessible channel formed in the Nqo1/NuoF/51-kDa (*Thermus*/*Aquifex*/*bovine* nomenclature) subunit. At the bottom of this channel, the FMN molecule, the primary electron acceptor for NADH, is located. As in many other dehydrogenases a specific motif, Rossman fold, participates in the binding of FMN and nucleotides. Rossman fold is unusually arranged in complex I. Instead of a six-stranded parallel β-sheet, it is represented by only four strands arranged in the order 4123 and flanked by α-helices. FMN molecule interacts with the loop connecting the 1st and 3rd strands and is adjacent to the 4th strand of β-sheet through a system of hydrogen bonds [20]. At the entrance to the nucleotide-binding channel of *A. aeolicus* enzyme, a conserved motif is located, including residues of phenylalanine Phe71 and tyrosine Tyr205 (in complex I from *T. thermophilus* a similar site is formed by two residues of Phe70 and Phe205 [39]), which, due to stacking interaction, stabilizes the adenine ring, providing a high affinity of complex I to NADH [39]. Adenosyl ribose is retained in the active center by hydrogen bonds with the glutamate residue Glu185 and the amino group of lysine Lys76. After initial binding, additional stabilization of the NADH molecule in a proper position occurs due to the formation of water-bridged hydrogen bonds between the phosphate residue associated with adenosyl ribose, Tyr205, and the adenine amino group [42]. The pyrophosphate moiety of the pyridine dinucleotide is not bound to the protein but interacts with the ribityl residue of flavin, ensuring its proper location. In this state, ribityl provides a water-bridged connection with Gly67 and Gly68 to bind the nicotinamide ribose. The nicotinamide ring is in water-bridges contact with the Glu95–Ser96–Glu97 triad and is additionally retained in the active center by stacking interaction with the FMN molecule [39,42]. In complex I of eukaryotic cells, this structure of the NADH-binding center is generally conserved [12].

**Figure 2:**
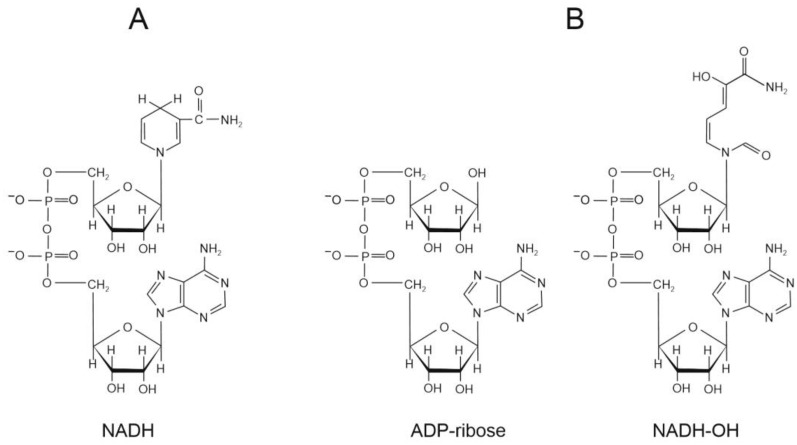
Structural formulas of NADH (**A**) and competitive inhibitors (**B**) interacting with the nucleotide-binding site of complex I.

In addition to the substrates NADH and NAD^+^, other nucleotides bind in the active site of complex I, including a reversible competitive inhibitor ADP-ribose [43] and NADH-OH interacting with an enzyme with high affinity [44] (Figure 2). Analysis of structures *A. aeolicus* complex I with bound substrates and inhibitors showed that the pyrophosphate and ribose attached to the adenine ring make the major contribution to the nucleotide binding, which in turn induces the proper location of the nicotinamide moiety relative to the isoalloxazine ring of FMN [42].

### 2.3. Redox Components of Complex I

**Figure 3:**
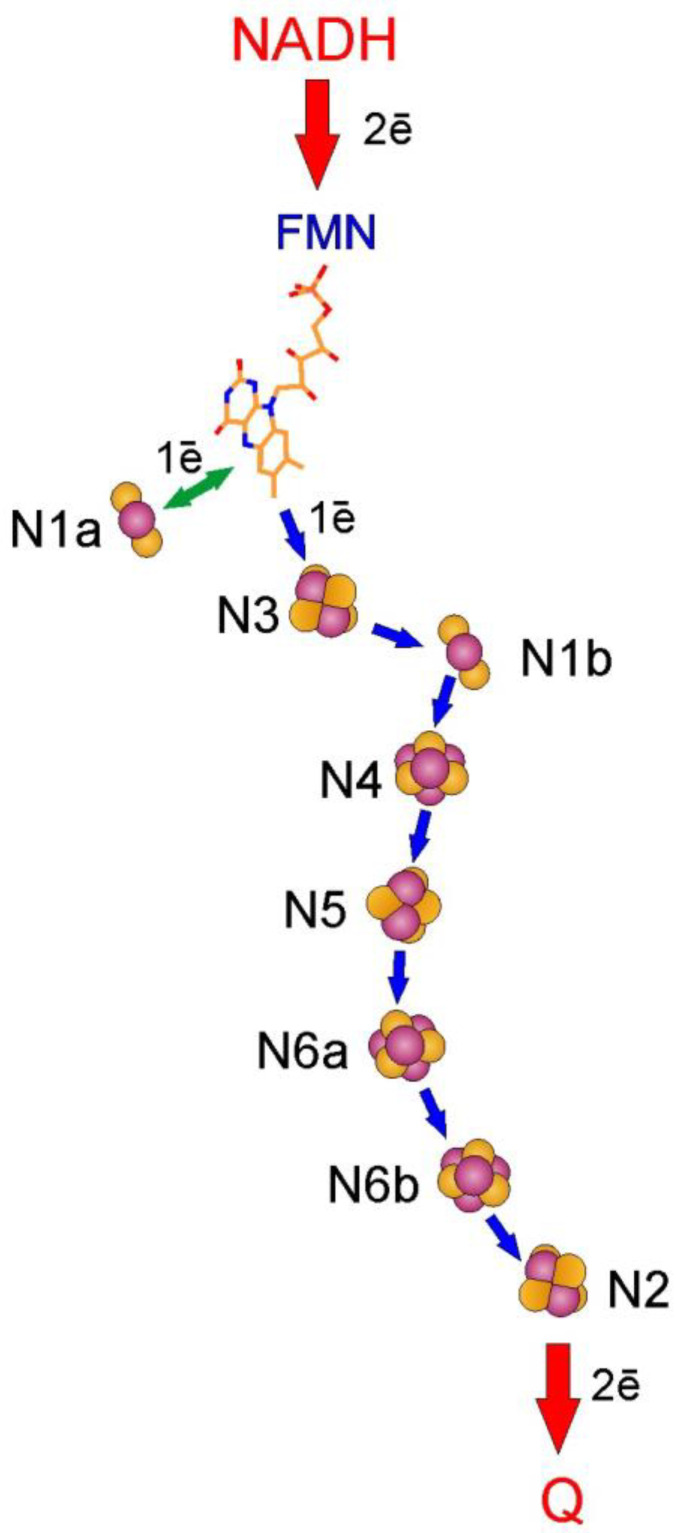
Arrangement of redox components of complex I. The main pathway of electron transfer from NADH to quinone (Q) is denoted with blue arrows. A diversion to cluster N1a is shown by a reversible green arrow. Two electrons from NADH reduce the FMN molecule. FMNH_2_ transfers one electron to the N3 cluster, and the second electron is transferred to the N1a center for “temporary storage”. After reoxidation of N3 by the next Fe-S cluster (N1b), N1a returns its electron back to FMN, which then transfers it to the N3 cluster.

Redox components of complex I participate in both catalyzing redox half-reactions and their coupling. During forward electron transfer, FMN is reduced by NADH in the PD-domain (on a µs timescale [45]) and then transfers those electrons along an approximately 80 Å long chain of 8 Fe-S clusters [46] to reduce Q to QH_2_ completing the redox reaction. Overall, there are 8–9 Fe-S clusters in complex I, two of which binuclear (N1a and N1b) while others are ferredoxin-type tetranuclear Fe-S clusters. Non-covalently bound FMN is the primary electron acceptor for a hydride anion (H^−^) from NADH, which is corroborated by FP (three subunit fragment solubilized from complex I upon chaotropic treatment) having diaphorase activities. Now-formed FMNH_2_ can only donate its electrons to nearby Fe-S clusters, which operate as strictly 1-electron (1ē) acceptors. From that follows that flavin’s oxidation should proceed sequentially through an FSQ**^•−^** radical (flavosemiquinone), which has indeed been observed by visible [47] and EPR (electron paramagnetic resonance) spectroscopy [48]. The arrangement of redox components of complex I is shown in Figure 3. The main pathway of electron transfer from NADH to Q depicted in Figure 3 is based on the structural information from the PD of *T. thermophilus* [46,49].

Potentiometric titration of FSQ**^•−^** in Hatefi’s preparation of bovine heart complex I revealed the midpoint redox potential for an overall 2ē oxidation to be around −375 mV at pH 7.5 (*E*_m,7.5_^2/0^ = −375 mV) with an expected pH dependence with a slope of −30 mV/pH [48]. Midpoint redox potentials for both sequential 1ē steps were also determined (*E*_m,7.5_^2/1^ = −336 mV for the first electron, and *E*_m,7.5_^1/0^ = −415 mV for the second one), suggesting radical stabilization observed as a small potential difference, which is similar to radical-stabilization properties of both complexes II and III [48]. Below pH 8.0, both 1ē steps have an expected pH-dependence of their midpoint redox potentials (−59 mV/pH), while at pH above 8.0, the second step becomes pH-independent due to almost complete dissociation of FSQH^•^ (protonated form of flavosemiquinone). The flavin radical signal displayed very fast relaxation rates and was not saturated in the power range assessed due to strong spin-spin interaction between the radical and Fe-S cluster N3 [48]. Guanidine (20 mM) was found to stabilize the flavin radical by increasing its stability constant more than 10-fold [48]. Qualitatively very similar results were obtained for FMN in FP fragment of bovine complex I by film voltammetry—the midpoint redox potential for an overall 2ē reduction of FMN was determined (*E*_m,7.0_^2/0^ = −401 mV) and displayed virtually the same pH-dependence [50]. Widths of peaks, corresponding to 2ē reduction of FMN, taken at half-heights, were comparable for the isolated enzyme (~80 mV [48]) and FP fragment (~85 mV [50]), which implies comparable FSQ**^•−^** stabilization in FP when compared to the whole enzyme. FMN bound to both the isolated complex I and FP fragment shows considerably more negative midpoint redox potential compared to free FMN in solution, which is a thermodynamic equivalent of a higher affinity of both enzymes to oxidized, rather than reduced, FMN.

Lower affinity of the enzyme to reduced FMN was exploited by showing reversible FMNH_2_ dissociation from complex I in bovine heart submitochondrial particles (SMPs) at alkaline pH (*K*_d_ ~10^−8^ M at pH 10.0 was reported) [51]. Recently, a potentially clinically significant loss of FMN in reducing conditions was observed in ex vivo neonatal mouse brain mitochondria following reperfusion after hypoxia–ischemia. In such conditions, complex I was fully reduced due to the dominance of succinate-supported reverse electron transfer (RET), which led to the reduction and dissociation of FMN [52]. What is interesting is that there was no FMN dissociation observed in such conditions when heart mitochondria were studied [53]. This difference was attributed to a brain-specific long isoform of subunit NDUFV3 affecting the affinity of the binding site to FMN [53].

Unsurprisingly, not only the affinity of the enzyme to FMN is redox state-dependent, but the affinity of the substrate-binding site to nucleotides as well. Reduced complex I has approximately 15 times higher *K*_d_ values for inhibitors ADP-ribose and NADH-OH [44], suggesting a reorganization of the substrate-binding site depending on the redox state of the enzyme. It was also shown [48] that when flavin radical is equilibrated using substrate redox pairs but not the redox mediator cocktail, the maximal FSQ**^•−^**/FMN_total_ ratio decreased from about 0.12 to 0.03, and it was now observed at around 100 mV more positive cell potential (−300 mV when compared to −400 mV for the mediator-equilibrated enzyme). The facts mentioned above as well as quantitative differences in FMN thermodynamic properties when bound to the whole enzyme and FP fragment suggest dynamic structure in the vicinity of FMN- and NADH-binding sites, which undergo redox-dependent changes triggering further conformational changes, which were observed upon complex I reduction and/or nucleotide binding using different approaches [54,55,56,57,58]. An extra line of evidence supporting such a view is the oxidative instability of redox components in FP fragments and alike [59].

FMN reduction by hydride anion could be described as a chemical transfer of electrons. This is not the electron transfer mechanism for the following steps. Upon reduction of FMN, there are two nearby Fe-S clusters, to which those electrons are presumably transferred (see Figure 3). FMN at first donates electrons to Fe-S clusters N3 and to an off-chain N1a in fast sequential reactions—estimated to be oxidized within 0.5 µs in the *E. coli* enzyme [45], and this is realized through electron tunneling, which is greatly enhanced by enzyme-bound water molecules [60,61]. We would like to stress the fact that polypeptide does not prevent tunneling but rather enables it, empty spaces are an obstacle. The common point of view [46] is that the electron that has arrived on the N3 cluster travels down the chain, while the N1a cluster now donates its electron back to FMN, from where it follows the former one. Fe-S clusters and their properties are not trivial to study, so a variety of approaches were used for that. Reduction in Fe-S clusters is usually monitored by EPR, and resulting spectra are not trivial in more than one sense—several individual signals can overlap and have different power saturation properties and relaxation rates. Signals originating from individual Fe-S clusters are named N1a, N1b, N2, N3, and so on. EPR studies on fragments of complex I and individual overexpressed subunits, combined with other approaches, have enabled assigning EPR signals to now structurally defined Fe-S clusters [46] (see Figure 3 for the original assignment by Ohnishi and coworkers, used in this paper; there is still debate in the field concerning the assignment of several of the signals [62,63]).

Fe-S clusters of complex I seem to be organized in a very effective way—electron tunneling between clusters is not a rate-limiting factor [64]. In most enzymes, electron tunneling occurs at edge-to-edge distances of 14 Å or lower, although it is a statistical reflection of the inverse relationship of electron tunneling rate constant on separating distance, not the universal law [65] meaning that 14 Å is not a physical distance limit for such transfers. As with other redox compounds, Fe-S clusters are characterized by their specific midpoint redox potentials, which are, though, quite complicated to unambiguously establish. This arises from significant electrostatic interactions between clusters [47]. Fe-S cluster N3, the immediate electron acceptor for FMN, is bound by a 51 kDa subunit (*bovine* nomenclature), while cluster N1a is by a 24 kDa subunit. These two clusters are also a part of the FP fragment of bovine complex I. Only signal N3 is observed upon reduction by NADH and Eu^II^-DTPA (*E*_m_ ~−1 V) in both preparations, while dithionite reduces N1a as well [66]. This seems to contradict the hypothesis of N1a being reduced by flavosemiquinone and is made even more difficult by the fact that the N1a signal is NADH-induced in *E. coli* [64]. Properties of signal N3 change upon dissociation of FP from the whole enzyme, although not radically [66]. In the whole enzyme, the following path of the electron is through sequential tunneling events through clusters N1b—N4—N5—N6a—N6b to the terminal cluster N2. Fe-S clusters that form the chain between FMN and N2 all have quite similar but not identical midpoint redox potential values of around −250 mV [67], which are influenced by the redox state of adjacent clusters [47]. Estimates were made that in conditions of steady-state electron transfer, 3–4 of all the enzyme’s Fe-S clusters are reduced [47,66]. From that follows that the redox state of an individual Fe-S cluster depends on its intrinsic midpoint redox potential, occupancy of clusters nearby as well as being affected by the protein surroundings. Such a viewpoint is corroborated by the fact that the intensity of signal N3 increases by around half when N4 is destroyed [68]. What is also of interest is the fact that clusters N1a, N2, N3, and N4 have different lability towards chemical modification. In the absence of NADH *p*-chloromercuribenzene sulfonic acid specifically destroyed signal N4, while in the presence of substrate, all signals were abolished; reduction by dithionite was not able to protect only cluster N4 from modification [68]. Again, cluster N4 is susceptible to N-bromosuccinimide treatment without a significant decrease in rotenone-sensitive NADH–decylubiquinone reductase, while both N3 and N4 signals were significantly reduced by diethylpyrocarbonate, a histidine reagent, along with all diaphorase activities [68]. From all the above follows that Fe-S clusters are accessible for small molecules and can be modified by and react with those while their accessibility and reactivity properties differ in more than one respect.

The terminal Fe-S cluster N2 is characterized by higher redox potential (around −140 mV in SMPs [67] and isolated complex I [69]) that is also shown to be pH-dependent (−60 mV/pH). A histidine residue in the 49 kDa subunit was shown to realize this effect in *Y. lipolytica* complex I [70]. The obvious potential gap between cluster N2 and preceding clusters as well pH dependence of its midpoint redox potential has long ago led to assumptions that events important for enzyme coupling take place in its vicinity. Fe-S cluster N2 is the donor of electrons for tightly bound ubiquinone judging by its strong spin-spin interaction with presumably ubisemiquinone radical (with a distance estimate of 8–11 Å [71]) and by the arrangement of Fe-S clusters in the enzyme [46].

Semiquinone radicals arising presumably from ubiquinone have been observed in complex I from bovine heart SMPs [71] and in prokaryotic enzymes [72,73]. Semiquinone signals in SMPs were *pmf*-dependent and were prevented by rotenone, suggesting the importance of a correctly organized ubiquinone-binding site for their appearance as well as a well-coupled membrane [71]. Two signals with significantly different relaxation times were originally detected suggesting a much tighter interaction for one of them. The origin of these signals was put in question recently with the use of an artificial vesicle-reconstituted chimeric respiratory chain, which was free from other respiratory chain enzymes [74]. Very low respiratory control values reported for those vesicles (1.25) could have precluded the formation of *pmf*-dependent semiquinone species, leaving the question open. Currently, the exact mechanism of ubiquinone reduction by complex I is not known. There are models suggesting concerted two-electron, two-proton-coupled reduction of ubiquinone by the N2 cluster with involvement of a nearby histidine as a proton donor as well as a tyrosine residue being a hydrogen atom donor [61] akin to cytochrome *c* oxidase, but there is new data that seem to contradict those [75].

### 2.4. The Q-Binding Center of Complex I

**Figure 4:**
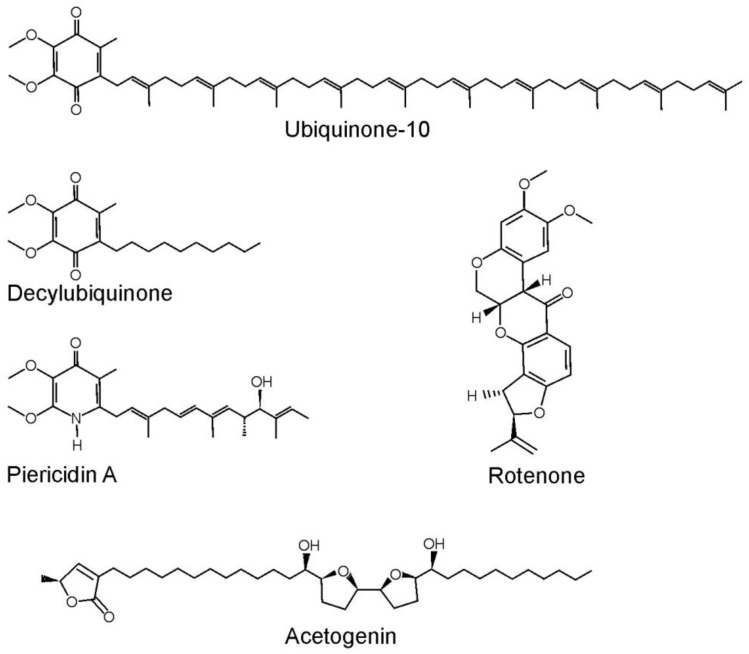
The structures of the substrates and the inhibitors of the ubiquinone-binding site.

The Q-binding center of complex I is localized between the PD and the MD. Two subunits of the Q-module, Nqo4 (49 kDa, *bovine* nomenclature) and Nqo6 (PSST), and Nqo8 (ND1) subunit of the MD participate in its formation [4]. This site has an unusual structure. It is a long (30 Å) and narrow channel, the entry point to which is located in the thickness of the membrane at the boundary with the phospholipid bilayer. The entrance gate is formed by one amphipathic and two transmembrane helices (TMHs) of Nqo8 and TMH of Nqo7 (ND3) subunits [4]. Three regions can be distinguished in the channel, differing in the properties of amino acid residues lining the Q-chamber. At the entrance, ubiquinone first interacts with hydrophobic residues of Nqo8 (ND1) and then it enters the channel from the phospholipid bilayer; it passes through the central highly hydrated region formed mainly by charged amino acids of Nqo8 and Nqo6, and then Q finally reaches the deepest amphipathic part formed by amino acids of Nqo4 and Nqo6. Here, the redox-active quinone ring (headgroup) is bound by two highly conserved residues Tyr87 and His38 of the Nqo4 polypeptide chain, which form hydrogen bonds with the carbonyl groups of the quinone headgroup [4]. In this position, the quinone headgroup is located at a distance of about 12 Å from the Fe-S cluster N2, the immediate electron donor for ubiquinone. The N2 cluster is deeply buried within the Nqo6 subunit, the distance from it to the membrane surface is approximately 15 Å [4]. The same structure of the Q-binding center has been confirmed in cryo-EM structures of complex I from other sources [10,12,14,76]. The length of ubiquinone-10 exceeds the size of the channel, so when the quinone headgroup reaches its top, the last 1–3 isoprenoid groups are situated outside the Q-cavity in the phospholipid bilayer [4]. A study of complex I structures with bound short-tailed quinones (decylubiquinone, dQ) (Figure 4) demonstrated that ubiquinone headgroup occupies two possible sites: one in the depth of the channel next to Fe-S cluster N2 at a distance of 12 Å, designated as Q_d_, and the other site, Q_s_, approximately in the center of the channel (24 Å from the entry to the channel) [4,77]. Although it is obvious that a natural long-chain ubiquinone will occupy the entire channel [11,16], the structures obtained with dQ [13,16,77] show possible sites of binding of the quinone headgroup as it moves deeper into the channel and during reverse movement into the phospholipid bilayer of the membrane. The putative intermediate sites of quinone headgroup binding, depending on the redox state of Q, as well as on conformational rearrangements in the surrounding region, are detected in the structure of the Q-chamber by molecular dynamics methods [78,79,80,81,82,83].

Inhibitory analysis remains a powerful tool for studying the mechanisms of interaction between enzymes and their substrates. Many inhibitors of a hydrophobic nature bind to the Q-binding site of complex I, among them the most known are rotenone and piericidin A [84,85] (Figure 4). As in the case of short-tailed ubiquinone analogs dQ, the binding of relatively short-length inhibitors that are not able to completely occupy the Q-channel occurs in two regions: in the depth of the channel (Q_d_-site) and in its central hydrophilic part (Q_s_-site), as has been demonstrated for complexes of the enzyme with piericidin A [4,77,86] and rotenone [13]. Cryo-EM structures of complex I with these inhibitors indicate possible intermediate stops of the ubiquinone headgroup as it moves into the cavity of the Q-binding channel, which corresponds to the data obtained by molecular modeling [78,79,80,81,82]. Acetogenins, strong natural inhibitors of complex I, and their synthetic analogs belong to another type of inhibitor. Their length allows them to occupy the entire cavity of the Q-channel. A molecule of an acetogenin contains a hydrophilic γ-lactone group associated with a 12-carbon aliphatic chain connecting γ-lactone with a central hydroxylated bis-tetrahydrofuran, which is in turn associated with a 10-carbon aliphatic terminal group [87] (Figure 4). This uniquely corresponds to the structure of the Q-channel: γ-lactone mimics the quinone headgroup, the central hydrophilic group occupies a place near the hydrophilic section of the channel, and two aliphatic parts of the molecule mimic the binding of the ubiquinone isoprenoid chain. The study of the cryo-EM structures of complex I bound with acetogenins led to the assumption that in the cavity of Q-channel, there are two energy minima for binding of the quinone headgroup: in the depth of the channel and in its central part. On the other hand, the presence of charged residues in the central part of the channel prevents the strong binding of the isoprenoid tail of ubiquinone and ensures its rapid movement deep into the channel and/or in the opposite direction, which in turn allows the catalysis of the NADH oxidation to happen with a high turnover [87].

Already in the pioneering cryo-EM structures of complex I, it was discovered that the mammalian enzyme can exist in two states [10,12], which were called “closed” and “open”. In the “closed” state, the inner space of the Q-chamber is formed by the β1-β2 loop of 49 kDa subunit (*bovine* nomenclature) and the loop of PSST subunit; TMH5-6 loop of ND1 is located in the lower part of the channel [13]. TMH2-3 of ND3 and TMH3-4 of ND6 are located at the interface between the PD and the MD. All loops are in an ordered state, which is clearly visible on cryo-EM structures. In the “open” state, the Q-channel expands, and the loops of 49 kDa, ND1, ND3, and ND6 subunits become disordered. A small loop (4 residues) in the PSST subunit, which protrudes from the channel in the “closed” state, unfolds, undergoing a “raised”—“flipped” transition, and a π-bulge appears in TMH3 of ND6 subunit [13]. During the transition to the “closed” state, re-ordering of these structures occurs, and the PD performs a lateral tilt and a slight twisting, which are clearly visible if you look at the top of the PD from the matrix side. As a result, the observed angle between the PD and the MD decreases. In the “open” state, the expanded Q-chamber ensures the penetration of ubiquinone, as well as various compounds from the phospholipid bilayer and a solvent (water) from the mitochondrial matrix into an inner cavity. In the “closed” state, the channel narrows and tightly covers the Q molecule, leaving only a small and narrow opening in the entrance area. Under these conditions, water access stops and the enzyme is ready for catalysis. Sazanov and colleagues consider the “closed” and “open” conformations of complex I to be intermediates of the catalytic cycle [13,88,89]. Hirst et al. proposed an alternative interpretation of the mixture of “closed” and “open” states of complex I identified by the cryo-EM method [90]. Their assumption is based on the fact that enzymes isolated from various mammalian species have significant differences in the ratio of “closed” and “open” structures, as well as a wide variety of structures characterized by an “open” conformation. Hirst and colleagues believe that the “closed” and “open” states of complex I are a reflection of the reversible transition discovered in the laboratory of Vinogradov and co-workers [91,92,93] between the active catalytically competent form of complex I and the deactive form of the enzyme, unable to catalyze the proton-translocating NADH–ubiquinone-reductase reaction (the two forms of complex I will be described in detail in Section 4). Under such consideration, the “closed” and “open” state of complex I can be attributed to the active and deactive forms of the enzyme with an ordered and disordered structure of the Q-binding center, respectively. Complex I catalyzes the NADH–ubiquinone-reductase reaction only in a “closed” conformation. The reorganization of the Q-binding center during the transition from the “open” to the “closed” state requires time much longer than the time of the catalytic turnover of the active enzyme [90]. A wide variety of cryo-EM structures obtained for enzymes from various sources with signs of an “open” state may reflect slow conformational changes in complex I during its transition to a deactivated form. In addition, only “closed” conformations are visible in the cryo-EM structures of complex I isolated from *M. musculus* and *P. denitrificans*. This, according to Hirst and co-authors, indicates that the “open” conformation cannot be intermediates of the catalytic cycle [5,14].

### 2.5. Membrane Domain of Complex I and Proton-Pumping Pathways

**Figure 5:**
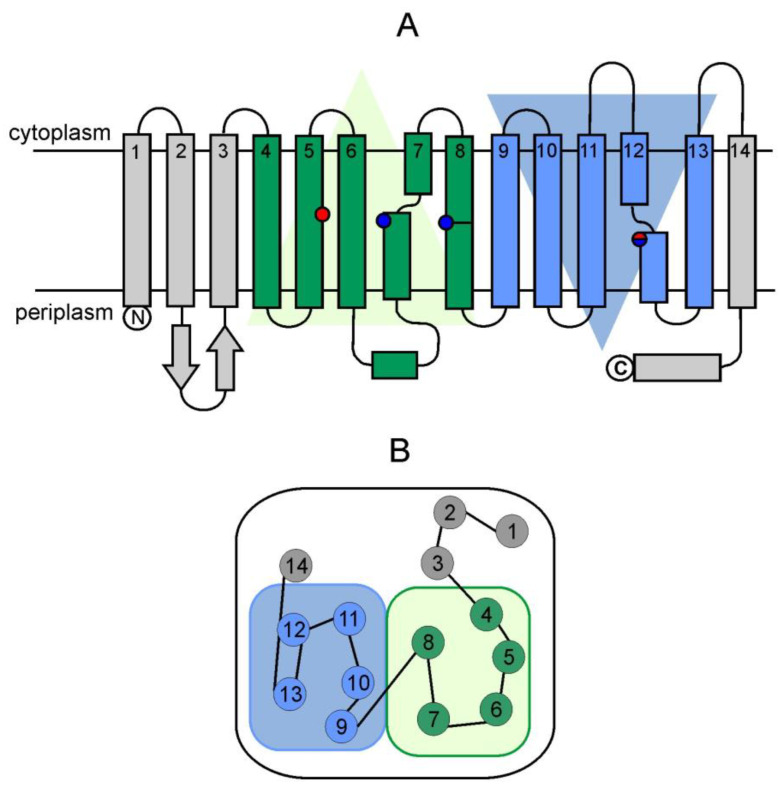
The architecture (**A**) and the arrangement of the N- and C-terminal half-channels in the ALS subunits ((**B**) view from the cytoplasm/matrix side). The N- and C-terminal TMHs’ repeats are colored in green and blue, respectively. TMHs are numbered with key residues indicated by circles in blue for Lys and in red for Glu.

The membrane domain of complex I includes 7 core subunits I (Table 1). Three large subunits, ND2, ND4, and ND5 (*bovine* nomenclature), have a homologous sequence and are assumed to participate in the vectorial transfer of three protons [4,26] because they are homologous to bacterial cation/H^+^ antiporters of the Mrp family [24,25]. Each antiporter-like subunit (ALS) consists of 14 transmembrane helices (TMH1–14) (Figure 5). Two conserved symmetric repeats (TMH4–8 and TMH9–13), but with an inverted orientation in the membrane, form putative half-channels for proton-pumping. The N- and C-terminal half-channels are exposed to the bacterial cytosol/mitochondrial matrix or to the periplasm/intermembrane space, respectively [26]. TMH7 and TMH12 are interrupted in the center by an extended loop of 5–7 residues containing conservative key LysTMH7/12 (or GluTMH12 in the ND4 subunit), which interacts with another conserved charged residues in the middle of the membrane and forms an entire transmembrane channel [26].

The key charged residues located in the center of the ALS form part of the long central hydrophilic axis passing through the ND2, ND4, ND5, and ND4L subunits approximately in the middle part of the membrane. It is assumed that the central axis provides long-distance communication along the MD [4,94]. Another element of the structure, a long amphipathic helix at the C-terminal site of the ND5 subunit, extending along the entire length of the membrane domain in close proximity to ND4 and ND2, presumably plays a structural role and does not participate in the coordination of proton transfer in the ALS subunits [95].

The oxidation of one NADH molecule by ubiquinone is accompanied by the transmembrane transfer of four protons [29,30]. Previously, it was assumed that each of the ALS pumps one proton [4,94]. The fourth putative channel in complex I is formed by the N1, N3, N6, and N4L subunits located at the interface between the PD and the MD. It contains key glutamate residues and is therefore called the E-channel [4].

It has recently been shown that among all the mentioned proton-translocating channels, only ND5 subunit has a full-fledged hydration profile providing a transport function [13,88]. These data were confirmed by the results of molecular dynamics simulations [38,96]. In the ND4 subunit, only the half-channel leading from the central axis to the cytosol/matrix is hydrated, whereas, in the ND2 subunit and the E-channel, both half-channels are “dry” (non-hydrated) and not capable of vectorial proton transfer. These data led to the assumption that all four protons are transported through a channel formed by the ND5 subunit. Despite the fact that the E-channel does not have access to either the cytosol/matrix or the periplasm/intermembrane space, it forms a controlled complete Grotthus-competent pathway for proton transfer from the Q-binding center to the central axis of complex I and thus plays an important role in the coupling of the redox reaction to transmembrane proton transfer [15,97].

In recent years, significant progress has been made in determining the structure of complex I, the most complex component of the respiratory chain. The structure of the enzyme’s active centers was characterized in detail, and various schemes for coupling the redox reaction catalyzed by complex I and transmembrane proton transfer were proposed [13,16,38,81,83,88]. However, these schemes are still hypothetical and the ways of their experimental verification are not yet visible. Due to modern high-tech technologies, new data are emerging that encourage researchers to refine, modify, and even revise the proposed schemes. It becomes clear that a wide arsenal of tools combining both studies of the structure and molecular dynamics of complex I and classical biophysical, biochemical, and molecular genetics approaches is required to elucidate the mechanism of the NADH–ubiquinone oxidoreductase reaction.

## 3. Interaction of Complex I with Artificial Electron Acceptors and Inhibitors

Complex I catalyzes the rapid oxidation of NADH by Q during its steady-state turnover. This reaction is well-known to be coupled to the transmembrane translocation of 4H^+^ per NADH [29]. In such conditions, even for an uncoupled enzyme (turnover rate of around 1000 s^−1^), dissociation of NAD^+^ from the active site is the rate-limiting factor [98]. Complex I is a fully reversible enzyme, so when *pmf*, reducing equivalents, and an electron acceptor are available the enzyme catalyzes RET—reverse electron transfer—ubiquinol-dependent *pmf*-supported reduction of the acceptor [93,98]. Upon the addition of rotenone, a specific tight inhibitor acting on the Q-binding site, electron transfer between Fe-S cluster N2 and ubiquinone stops, which leads to the inhibition of NADH oxidation and decoupling of redox activities from the proton pumping. Under such conditions, which are functionally similar to the D-form of the mammalian enzyme and thus model an ischemia–reperfusion injury state, electron transfer between FMN and Fe-S clusters is not blocked, as well as FMN reduction by NADH in the substrate-binding site. The enzyme is reduced by NADH and can be oxidized by a variety of artificial electron acceptors, thus giving rise to diaphorase activities. It turns out that FMN can provide enough NADH oxidation to support much higher turnover rates—more than 10,000 s^−1^ when hexaammineruthenium (HAR) is the acceptor [99].

Tight *pmf*-independent binding of rotenone (*K*_i_ of around 1 nM) not only leads to inhibition of the ubiquinone reduction but also changes the equilibrium between A- and D-forms of the enzyme shifting it towards active complex I [100]. The dissociation constant for the enzyme–inhibitor complex was shown to be 20 times higher when RET to NAD^+^ was measured, suggesting redox influences on the rotenone binding. Rotenone is the commonly used specific inhibitor, but not the only substance that severes the connection between Fe-S cluster N2 and ubiquinone. For example, routinely used detergent Triton X-100 realizes the same inhibitory effect with an apparent *K*_i_ of 1 × 10^−5^ M [101], while there are many more known compounds of different chemical nature acting on ubiquinone-binding site [84]. These facts coupled with the expanding use of detergents for complex I purification and solubilization require caution and attention when both setting experiments up as well as interpreting data.

Complex I reduced by NADH or by succinate in RET can be oxidized by oxygen, ferricyanide (FC), and HAR [98]. The first of these reactions can no doubt occur in vivo, so it is of obvious interest. There are two obvious possibilities for complex I to be oxidized by oxygen—flavin and ubiquinone radicals are known to do exactly that. Currently, the consensus seems to be that only flavin radical is involved in this reaction [102,103]. Thus, molecular oxygen accepts an electron becoming a superoxide radical, which gives rise to other ROS. This ROS generation by complex I is stimulated by rotenone and RET conditions, which is expected since leading to a more reduced enzyme state [102]. What is important is that ROS production by complex I is severely inhibited by NAD^+^ and NADH—the optimal activity was revealed in the presence of only 50 µM NADH while at 1 mM substrate concentration, the activity was 5 times lower [102]. What is striking is that 0.1 mM NAD^+^ inhibited both NADH- and RET-supported ROS production of complex I by 80%, which has probably happened due to the enzyme now also catalyzing transhydrogenase reaction [102]. We point out that in the mitochondrial matrix, NAD(H) concentration is in a millimolar range, which is expected to inhibit ROS production by complex I. It was also found that glutamic acid residue (Glu95 NuoF), situated in the vicinity of FMN, limits ROS production by *E. coli* complex I—the E95Q mutant had much higher activity [1]. The optimal experimental conditions for ROS generation by complex I in SMPs were found to be during succinate-supported RET as well as with rotenone-inhibited enzyme at low substrate concentrations; but even in such cases, the activity is only around 1 nmol NADH oxidized per min per mg, which corresponds to several percent of the steady-state coupled electron flow [104].

**Figure 6:**
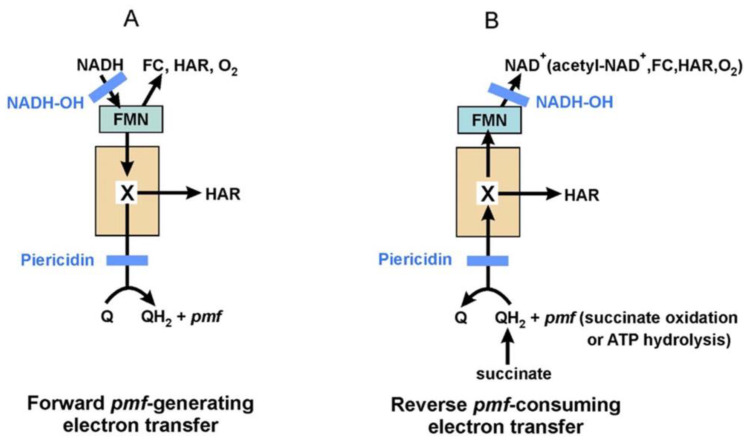
Proposed scheme of electron-transfer pathways in complex I during forward (**A**) and reverse reactions (**B**). See the text for an explanation.

Of nucleotides, there are much better inhibitors of complex I-mediated ROS production—ADP-ribose and NADH-OH. Both of these substances are breakdown derivatives of NADH. ADP-ribose is a classic competitive reversible inhibitor with *K*_i_ of around 25 µM for both bovine and *P. denitrificans* enzymes [43,105]. NADH-OH, though, is an inhibitor with much tighter binding—*K*_i_ of 3 × 10^−10^ M for the oxidized bovine complex I and *K*_i_ of 1 × 10^−9^ M for *P. denitrificans* enzyme were reported [44,105]. Both of these inhibitors show pronounced unidirectional effect on complex I activities—dissociation constants for enzyme–inhibitor complexes are much higher in RET than during forward electron transfer, which is a manifestation of redox-dependent changes in the nucleotide-binding site [44]. NADH-OH is a particularly useful inhibitor since it completely suppresses all NAD(H)-dependent reactions of complex I as well as RET to oxygen and FC in very low concentrations [41,44,99,105] (see scheme in Figure 6). It was shown recently that tight binding of NADH-OH is realized through two additional hydrogen bonds that stabilize its amide group in the NADH-binding pocket [41]. The authors concluded that binding of the inhibitor leaves no space available even for oxygen to reach FMN [41].

FC is the first established “efficient” artificial electron acceptor for complex I. In classic works by Dooijewaard and Slater, it was found that NADH–FC-reductase activity of complex I proceeds via a ping–pong bi–bi mechanism characterized by pronounced double substrate inhibition [106]. It seems that, currently, some researchers in the field treat ping–pong mechanisms as a strict indication of the necessity of NAD^+^ dissociation before subsequent acceptor reduction. We would like to point out that this is not the only fitting interpretation. Such a kinetic mechanism, otherwise called double-displacement or *enzyme*-displacement, arises when and if there is a *kinetically* irreversible step preceding the reaction with the second substrate (FC in this case). Not only NAD^+^ dissociation but also exergonic intramolecular electron transfer events are kinetically irreversible during steady-state initial rate measurements.

Upon dissociation of FP fragment from bovine complex I, its NADH–FC-reductase activity changes drastically. The reaction now proceeds via a combined ordered and ping–pong bi–bi mechanism, which depends on FC concentration (shifting more towards an ordered mechanism with an increase in the substrate concentration) [107]. This, again, shows that the structure of FP differs from that of the intact enzyme. Also, it follows from above that FMN is the most probable electron donor for negatively charged FC as well as for molecular oxygen. FC can be reduced not only by NADH but also in RET by coupled bovine heart SMPs and *P. denitrificans* plasma membrane vesicles [91,105], which presents simple Michaelis kinetics. This means that the rather straightforward interaction of the reduced enzyme with the acceptor is significantly influenced by the presence of substrate.

HAR is a positively charged complex compound with a midpoint redox potential around 0 mV that is auto-oxidizable by molecular oxygen in a slow second-order nonenzymatic reaction upon reduction, while at the same time also being a good substrate for cytochrome *c* oxidase [108,109,110]. It was introduced in practice as the only electron acceptor that had its turnover rates scale with the FMN content of preparations. This coupled with the absence of any substrate inhibition has made it a great tool for purification control during isolation procedures. NADH–HAR-reductase activities of complex I in SMPs and *P. denitrificans* “subbacterial particles” (SBPs; plasma membrane vesicles) look simple and similar at first—there is an apparent saturation of activity when increasing concentrations of any of the substrates and the reaction seems to follow strictly ordered mechanism (ternary complex) [105,111]. On a closer inspection, it turned out that at low substrate concentrations, bacterial enzyme switches to a ping–pong bi–bi mechanism [105] reminiscent of the bovine FP fragment interaction with FC.

FP fragment of bovine complex I also catalyzes NADH–HAR-reductase activity, but it is also quite different from the activity catalyzed by the whole enzyme as in the case of FC. Double reciprocal plots of activity vs. HAR concentration were not actually linear suggesting an absence of real saturation [112]. Also for FP, it was shown that FC reduction was stimulated by 50 mM guanidine, while HAR reduction was inhibited at higher NADH concentrations in a competitive manner, and activated at concentrations lower than 25 µM [112]. This was interpreted as HAR interacting with negatively charged residues/surfaces and thus being screened by another cation.

NADH–HAR-reductase activities of bovine heart SMPs and of Hatefi’s isolated complex I are identical. The whole bovine enzyme always operates via a ternary complex mechanism [111], and the reaction is completely blocked by NADH-OH [44] (this is also the case for *P. denitrificans* enzyme [105]). When we were accessing inhibition of NADH–HAR-reductase activity by ADP-ribose, a competitive inhibitor, we found that at low substrate concentrations, the compound was an activator [113]. It led to the identification of free ATP as an effective activator (up to 10-fold activation depending on substrate concentrations) of bovine complex I NADH–HAR-reductase activity with an apparent *K*_a_ of around 20 µM. Neither FP nor bacterial complex I demonstrated this activation by ATP in a wide range of substrate and effector concentrations studied, while SMPs extracted from *Y. lipolytica* did [113]. That showed not only effective ATP binding to complex I but is also one of the few known kinetical differences between eukaryotic and prokaryotic enzymes. We have interpreted these results as ATP-induced changes to redox components of the enzyme [113], while Hirst and coworkers have suggested a unified reaction scheme that explains ATP-induced activation as being realized through the introduction of negatively charged phosphates into the vicinity of NADH-binding site [113,114]. A similar reaction mechanism was proposed for another positively charged compound—paraquat [114]. What is interesting is that NADH–HAR-reductase activity of *Y. lipolytica* complex I mutant (A341V in the homolog of 51 kDa subunit) was completely abolished while not affecting the reaction with paraquat, actually suggesting different mechanisms for reduction of these two compounds [115]. The mutated amino acid residue is located close to FMN but is not facing it, rather pointing to the outside. The former and the fact that the mutation results in a chemically and sterically very similar residue makes us state that even slight changes to local surrounding of redox components could have profound effects on interaction of complex I with HAR.

Recently we have shown that bovine heart SMPs and *P. denitrificans* SBPs catalyze RET on HAR. Activities of both enzymes were sensitive to uncouplers, rotenone/piericidin A and inhibition/dissipation of *pmf* generation. What is important is that for both enzymes RET on HAR was not blocked by an excess of NADH-OH, which was shown to inhibit all other activities (NAD(H) interaction and reduction of FC and oxygen) in exactly the same conditions [99,105] (Figure 6). So, in essence, we have observed FMN-independent RET to HAR in both mammalian and bacterial enzymes. All of the above leads us to question the currently accepted view of only FMN being a redox-active group of the enzyme in terms of interaction with hydrophilic substances. The fact that NADH-OH-inhibited complex I reduces HAR implies the existence of another electron transfer route since access to the FMN-binding cavity is completely blocked [41]. As was mentioned above, Fe-S clusters of complex I are differentially accessible for chemical modification by different compounds [68], have their redox properties linked to redox states of nearby components [47,66], and change their properties upon dissociation of the enzyme into subcomplexes. In summary, we do not see any contradictions to the possibility of a Fe-S cluster serving as an electron donor for an artificial electron acceptor; rather, this point of view explains the insensitivity of RET on HAR to NADH-OH. If such a possibility is in fact realized, it then opens new and interesting areas of research—NADH-independent reduction of the enzyme is an intriguing analytical possibility, while the potential physiological significance of presumed FMN-independent electron flow through an enzyme could also be checked.

## 4. The Slow Active/Deactive State Enzyme Transformation (A/D Transition)

**Figure 7:**
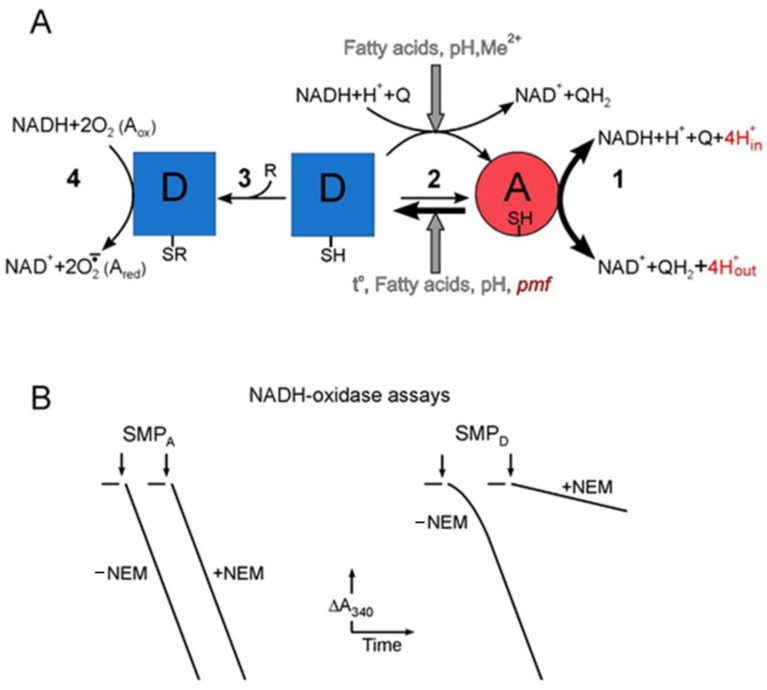
Scheme illustrating the slow A/D transition of the mitochondrial complex I. (**A**) Reaction (1). The active complex I (A-form) catalyzes rapid NADH–ubiquinone (Q) reductase reaction coupled with vectorial translocation of 4 protons (*pmf* generation). Reactions (2). In the absence of substrates, a slow spontaneous transformation of the A-form into the D-form (D) occurs and equilibrium is established. The rate of A→D transition (deactivation) increases in the presence of fatty acids (FA) and at higher pH and slows down with energization of the mitochondrial membrane (*pmf* generation). Turnover-dependent D→A transition (activation) is shown; its rate is decreased by fatty acids, divalent cations (Me^2+^), and at the higher pH. Reaction (3). The SH group (Cys39 of the ND3 subunit) in the D-form becomes accessible for thiol-reactive reagents (R) and is irreversibly modified. Reaction (4). Both D-forms shown are capable of catalyzing the oxidation of NADH by artificial electron acceptors (A_ox_) or molecular oxygen with ROS formation. (**B**) The A-form of complex I in submitochondrial particles (SMP_A_), control (–NEM), or modified by N-ethylmaleimide (+NEM) catalyzes NADH oxidation in the zero order (left panel). The D-form (SMP_D_) oxidizes NADH with a lag phase. SMP_D_ preincubated with NEM is unable to catalyze the NADH oxidase reaction (right panel).

Complex I plays an extremely important role in the metabolism of a living cell. As already noted, it not only creates a driving force for ATP synthesis but also maintains a certain ratio of the NAD^+^/NADH redox pair necessary for an efficient turnover of central catabolic pathways. Currently, little is known about the mechanisms of fine regulation of this enzyme. The role of supernumerary subunits in complex I in regulating its activity is widely discussed in the literature, but their participation in this process remains unclear [28]. One of the ways to regulate complex I can be a phenomenon described more than 30 years ago in Vinogradov’s laboratory and formulated as a hypothesis about the existence of two forms of complex I [91,93,116] (scheme in Figure 7). The active A-form catalyzes the reversible NADH–ubiquinone-reductase reaction coupled with transmembrane transfer of four protons and *pmf* generation with a high turnover number. In the absence of substrates (NADH and oxidized ubiquinone), the A-form slowly transforms into an inactive (deactivated) D-form, which is unable to catalyze the reduction of ubiquinone but still catalyzes the oxidation of NADH by artificial electron acceptors (FC, HAR, O_2_, etc.). The A/D transition is an equilibrium process. The rate of deactivation (transformation of the A-form into the D-form) strongly depends on temperature and proceeds at relatively high temperatures, above 30 °C. The temperature, however, does not affect the equilibrium, which is strongly shifted towards the formation of the D-form, the proportion of which is about 90% in equilibrium [117]. The activation barrier of the A→D transition is 270 kJ/mol, which indicates significant rearrangements in the complex I molecule. It can be assumed that during deactivation, complex I transforms into a stable (resting) state at the minimum of its potential energy [116]. In addition to temperature, deactivation is strongly affected by free fatty acids, which increase the rate of transition of complex I to the D-form [118]. The rate of deactivation, being pH dependent, is greatly increased at the higher pH [117]. When ubiquinone in the respiratory chain is oxidized, the addition of NADH leads to the transformation of the D-form into the A-form as a result of one or more slow catalytic turnovers [91,93,116]. The activation process is clearly visible when the NADH oxidase or NADH–ubiquinone oxidoreductase reaction is registered: A pronounced lag phase occurs, then, the rate of NADH oxidase reaches a stationary level and does not change further (Figure 7B) [91]. The lag phase is completely absent when the reaction is catalyzed by a fully active complex I, represented only by the A-form. The A-form, apparently, is not energetically stable, since the absence of substrates again leads to the appearance of the D-form of the enzyme, and such mutual transitions can be repeated many times [116]. This fact indicates that during catalysis, part of the free energy released during the oxidation of NADH by ubiquinone is spent on maintaining the catalytically competent structure of complex I [116]. The rate of D→A transition is strongly inhibited by divalent cations [119], fatty acids [118], and the higher pH [119], while the reaction rate in the stationary state, when complex I is completely in the A-form, does not depend on pH in the range 6.5–8.5 and is not affected by divalent cations. EPR spectroscopy of complex I showed that deactivation affects only the ubiquinone-binding part of the enzyme. When NADH is added to the D-form, all iron–sulfur centers of complex I are rapidly reduced [120].

The A- and D-forms differ not only functionally but also structurally. It has been shown that the A-form is not inhibited by thiol-reactive reagents—N-ethylmaleimide (NEM), *p*-chloromercuribenzoate, and 5,5′-dithiobis-(2-nitrobenzoic acid)—even for long incubation, whereas Cys39 in the ND3 subunit of D-form becomes accessible for modification and binds to NEM [121]. Irreversibly modified D-form is no longer able to transform to active A-form. The sensitivity of complex I to thiol-reactive reagents is a diagnostic test of deactivated enzyme. On the other hand, the D-form does not interact well with rotenone, a specific inhibitor of the Q-binding center; the dissociation constant of the enzyme complex with rotenone is decreased by two orders of magnitude compared with the A-form [100].

The A/D transition was detected in an isolated mammalian enzyme, SMPs, intact mitochondria, and perfused hearts [116,122,123]. Notably, the A/D transition is absent in bacteria and non-vertebrates. In cold-blooded vertebrates and fungi (*Y. lypolitica* and *Neurospora crassa*), the transition of the A-form to the D-form occurs at relatively low temperatures, in which these organisms live; this indicates a low activation barrier of the A/D transition, as well as its possible physiological role [116,122,124].

In the cryo-EM structures of the D-form, there are no “closed” states and a large-scale reorganization is observed, primarily expressed in the structure of the Q-binding center. A strong tilt of the TMH4 of the subunit ND6 can be seen; this is accompanied by a disordering of the TMH3-4 loop and its insertion between the PD and the MD domains, which prevents the reverse transition to the A-form. The unfolding of the β-sheet in the ND6 subunit and pronounced π-bulge on the TMH3 are visible on the structures. Disordering extends to areas around the amphipathic helix of the C-terminal of the antiporter subunit ND5 and is observed in some additional subunits located outside the Q-binding site. The destructurization of the TMH1-2 loop of ND3 leads to the accessibility of the Cys39 to the solvent and consequently to the thiol-reactive reagents [13,90]. Such significant structural changes in the complex I molecule reflect the high activation barrier of the A/D transition. Notably, the A- and D-forms were detected in complex I isolated from bovine hearts subjected to ischemia [125].

Incubation of the deactivated complex I in SMPs with rotenone led to the appearance of a significant fraction of the A-form of the enzyme. It has been suggested that the tight binding of rotenone causes the same conformational changes that occur when ubiquinone binds to the Q-site in the catalytic cycle, and the enzyme–inhibitor complex likely falls into a transition-state analog [100]. This assumption contradicted the generally accepted view that rotenone, whose structure is not similar to ubiquinone (Figure 4), cannot bind in the Q site. However, recent cryo-EM structures of complex I with bound rotenone have demonstrated that the inhibitor binds in the depth of the Q-site (Q_d_-center) and mimics the conformation of bound quinone during its reduction by the N2 cluster [13]. The rotenone is a flexible molecule and can exist in two different conformations, bent and straight. For passage through the Q-channel, a straight formation is optimal, and for tight binding in the depth of the channel, a bent one is optimal. Recently, a dehydrated rotenone has been synthesized, which is represented by a single straight conformer. Dehydrated rotenone inhibited complex I with 600-fold less potency than natural rotenone [126]. These data directly demonstrate that when the rotenone reaches the Q_d_-site, it changes its structure, transforming into a bent conformer, which leads to its strong binding. Thus, the enzyme complex with rotenone can indeed be considered as a transition-state analog [100].

The possible physiological significance of the A/D transition is currently unclear. It can be assumed that a large number of components of the mitochondrial matrix, both of a protein and non-protein nature, can, on the one hand, directly affect the catalytic activity of complex I, and on the other hand, affect the rates of transition and the equilibrium between the A- and D-forms of complex I, thus regulating the rate of electron transfer in the respiratory chain. Ex vivo experiments have shown that normally about 5–15% of complex I in the heart is in D-form [127,128]. Perhaps this part of the enzyme represents a reserve of activity that can be used under changing conditions, such as reductive stress, changes in ATP demand, and oxygen availability [122]. Recently, it was shown in our laboratory that the energization of bovine heart SMPs by ATP hydrolysis decreases the rate of complex I deactivation [117]. This is the first indication of the possible role of *pmf* in the regulation of complex I activity.

Any imbalance between two forms of complex I and the full transformation to a deactivated state can lead to pathophysiological consequences. In ischemia, under conditions when the oxygen concentration is dramatically decreased, the respiratory chain is completely reduced, and complex I, in accordance with the thermodynamics of the A/D-transition, will transform into a D-form, unable to transfer electrons to the respiratory chain, but potentially capable of ROS generating. Subsequent re-oxygenation will be dangerous, since when oxygen enters the cells, the D-form will not be able to transfer electrons downstream into the respiratory chain but will actively produce ROS, which may have a negative consequence due to damage to many cellular structures [116]. Another scenario considers the D-form of complex I as a protection against a burst in ROS generation during re-oxygenation; since at the early stage of oxygenation, the D-form is unable to catalyze RET from succinate accumulated during ischemia [122]. Keep in mind that RET, catalyzed by complex I in intact mitochondria, is the main source of ROS [129]. Currently, the relative contribution of ROS produced by complex I to damage the cellular structures is still debated [130,131,132,133,134,135].

## 5. Biomedical Aspects

Complex I deficiency is the most frequently encountered single enzyme deficiency that causes mitochondrial disorders. Pathogenic mutations were identified in most of the mtDNA and nDNA genes encoding structural subunits of the enzyme, and the genes encoding assembly factors. The most frequently reported core clinical features associated with the mutations are leukodystrophy (Leigh syndrome), cardiomyopathy, Leber optic atrophy, fatal lactic acidosis, encephalopathy, and microphthalmia with linear skin defects [3]. Complex I mutations can lead to both a decrease in the enzyme activity and a number of secondary effects at the cellular level, including increased ROS production, altered membrane potential, mitochondrial morphology, and intracellular calcium homeostasis [3].

Notably, in hypoxia, certain cancer cells reveal residual complex I activity that oxidizes NADH. The resulting NAD^+^ is needed for α-ketoglutarate dehydrogenase complex, a key enzyme in the oxidative decarboxylation branch of glutaminolysis—a hallmark oncometabolic pathway [136]. Succinyl-CoA produced by α-ketoglutarate dehydrogenase complex is then used by succinyl-CoA synthetase to support mitochondrial substrate-level phosphorylation. This partially compensates for an oxidative phosphorylation deficiency in cancer cells in the absence of O_2_. The necessity of complex I in this process provides a rationale for exploring the enzyme inhibitors in cancer treatment [136]. Consistently, the inhibition of complex I was reported to slow tumor growth under a few experimental settings [137,138,139,140,141,142]. To date, drug toxicity seems to be the main obstacle in using highly potent and selective inhibitors of this respiratory enzyme against cancer designed so far [136]. However, an encouraging preprint has been published recently showing that Fasnall, an inhibitor of fatty acid synthase in cancer cells, inhibits complex I and impairs tumor growth [143]. Importantly, Fasnall-treated mice do not reveal neurological side effects [143], which were reported for some other inhibitors of complex I [144]. Thus, the potential use of complex I as an anti-cancer target sounds promising.

Using cryo-EM and enzyme kinetics, Bridges et al. [145] gained molecular insights into the inhibitory mechanisms of biguanides on mammalian complex I catalysis. The biguanide metformin, widely prescribed for type 2 diabetes, targets a number of cellular proteins, including complex I. Inhibition of complex I by metformin is suggested to lead to a decrease in ATP synthesis that in turn causes inhibition of hepatic gluconeogenesis [146]. Bridges et al. used IM1761092, a more hydrophobic derivative of metformin [145]. IM1761092 shows a much stronger inhibition of complex I activity in bovine heart mitochondrial membranes than metformin, giving IC50 in the low micromolar range. Like antidiabetic biguanides, IM1761092 inhibits catalysis via binding to the deactive state of the enzyme and the inhibitory effect is stronger at higher pH values. There are three biguanide-binding sites in complex I, which are independent of one other. Site 1, the primary inhibitory site, is an amphipathic region of the quinone-binding channel (Q-channel) that straddles two zones of the channel, the hydrophobic region near the exit and its charged central region. This region probably becomes exposed to the matrix in the deactive state and adjacent to the mobile element in NDUFS7 (human nomenclature). The latter element carries Arg77 and switches the Q-channel conformation between the active and deactive states. Site 2 is at the subunit ND5 lateral helix–subunit NDUFB4 interface. Site 3 is in a pocket formed by subunits ND2, NDUFB5, and NDUFA11 (*human* nomenclature) on the intermembrane-space side of complex I. The interaction of IM1761092 with site 3 is suggested to be noninhibitory. Thus, biguanides implement their inhibitory mechanism mainly via the major interaction site located inside the Q-channel by preventing reactivation of the resting deactive state. The deactive state of the enzyme forms during oxygen starvation, e.g., during ischemia and in solid tumor microenvironments. Unlike canonical inhibitors, discrimination of biguanides for the deactive state allows them to selectively target hypoxic tissues with less risk of compromising respiration in tissues containing the active complex I. By targeting the deactive state biguanides could also reduce ROS production by the enzyme in the active state by means of RET. The report by Bridges et al. [145] offers a basis for future structure-based drug design in the development of biguanide-based therapies for diverse therapeutic applications, including cancer treatment.

Energy metabolism of pathogenic microorganisms is becoming an area that shows promise for the development of next-generation antimicrobials [147,148,149,150]. Type 1 and type 2 NADH dehydrogenases of microbial respiratory chains (also named as NDH-1 or complex I and NDH-2, respectively) are among potential protein drug targets. In this regard, a few recent reports are worthy of mention. In particular, Lettl et al. reported [151] that *Helicobacter pylori* is extremely susceptible to small-molecule complex I inhibitors, such as diflumetorim, fenpyroximate, and fenazaquin derivatives, whereas intestinal bacteria remain unaffected. *H. pylori* is known to be a human gastric pathogen associated with such diseases as gastric cancer, atrophic gastritis, and peptic ulcer disease. The peculiarity of *H. pylori* complex I is that it probably uses flavodoxin rather than NADH as the electron donor, and menaquinone instead of ubiquinone as the electron acceptor. Based on molecular modeling, phenotypic assays, and selection of resistance-inducing mutations, the observed inhibitor hypersensitivity is explained by a unique quinone-binding pocket formed by NuoB, NuoD, and NuoH subunits. The pocket has a narrow entry channel and a spacious inner cavity with a quinone reduction site close to Fe-S cluster N2 and a second quinone-binding site. The modeling data suggest the potential binding of inhibitory compounds in either binding site. The authors concluded that the complex I inhibitors can serve as candidates for the development of species-specific antimicrobial agents against *H. pylori*. This conclusion is supported by the fact that the compounds reveal no cross-resistance with currently used eradication therapy drugs, do not speed the development of resistance, and do not have a significant effect on the gut microbiota [151].

Recently, Lee et al. showed that N-(4(4(trifluoromethyl)phenoxy)phenyl)quinazolin-4-amine (ND-011992) targets a mycobacterial *bd*-type quinol oxidase [152]. Using deamino-NADH (d-NADH) as a substrate-specific for complex I, Kagi et al. [153] found that ND-011992 is a potent inhibitor of the complex I in the *E. coli* membranes, giving IC50 of 0.12 μM. It is worth mentioning that ND-011992 also inhibits the *E. coli* quinol oxidases *bd*-I, *bd*-II, and *bo*_3_ but with less efficiency. The respective IC50 values for the isolated enzymes are 0.63, 1.3, and 2.47 μM. Note that these types of terminal respiratory oxidases are present in bacteria, including pathogenic species, but absent in the mitochondria of humans and animals [154]. Importantly, inhibition of complex I in the membranes from bovine heart mitochondria turned out to be about 27-fold lower than that of the *E. coli* complex I [153]. Thus, ND-011992 could be a starting material to develop derivatives that would specifically inhibit a bacterial complex I per se or even the entire bacterial respiratory chain, without significantly affecting the eukaryotic counterparts.

## 6. Concluding Remarks

Complex I is the complicated component of the respiratory chain of mitochondria and the plasma membrane of bacteria. It plays an important role in the metabolism of living cells, as it provides a driving force for the synthesis of ATP in oxidative phosphorylation. In recent years, significant progress has been made in resolving the structure of complex I, and hypothetical mechanisms for coupling the redox reaction to transmembrane proton transfer have been proposed. Currently, it is necessary to focus on the efforts of the world’s leading laboratories to experimentally prove these hypotheses. To achieve this goal, it is necessary to use both new high-tech methods and classical biophysical, biochemical, and molecular biological research approaches. Solving the mechanism of complex I will be crucial not only for fundamental science but also for the introduction of new knowledge into theoretical and practical medicine. Targeting the human complex I offers opportunities for therapeutic intervention in a number of diseases, including cancer. NADH–quinone oxidoreductase of pathogenic microorganisms can be considered as attractive target in antimicrobial drug discovery.

## Figures and Tables

**Table 1:** Nomenclature of core subunits of complex I from various sources.

Bacteria		Fungi	Insects	Mammals	
*T. thermophilus* *P. denitrificans*	*E. coli* *Aqiflex aeolicus* *Rodobacter capsulatus*	*Y. lipolytica*	*D. melanogaster*	*B. taurus* *O. aries* *M. musculus* *S. scrofa*	*Homo sapiens*
Peripheral domain N-module (dehydrogenating)					
Nqo1	NuoF	NDUFV1	NDUFV1	51 kDa	NDUFV1
Nqo2	NuoE	NDUFV2	NDUFV2	24 kDa	NDUFV2
Nqo3	NuoG	NDUFS1	NDUFS1	75 kDa	NDUFS1
Q-module (connecting)					
Nqo4	NuoD ^a^	NDUFS2	NDUFS2	49 kDa	NDUFS2
Nqo5	NuoC ^a^	NDUFS3	NDUFS3	30 kDa	NDUFS3
Nqo6	NuoB	NDUFS7	NDUFS7	PSST	NDUFS7
Nqo9	NuoI	NDUFS8	NDUFS8	TYKY	NDUFS8
Membrane domain					
P-module (proton-translocating)					
Nqo7	NuoA	ND3	ND3	ND3	ND3
Nqo8	NuoH	ND1	ND1	ND1	ND1
Nqo10	NuoJ	ND6	ND6	ND6	ND6
Nqo11	NuoK	ND4L	ND4L	ND4L	ND4L
Nqo12	NuoL	ND5	ND5	ND5	ND5
Nqo13	NuoM	ND4	ND4	ND4	ND4
Nqo14	NuoN	ND2	ND2	ND2	ND2

^a^ In complex I of some bacteria, for example, in *E. coli*, NuoD and NuoC subunits are fused into a single polypeptide chain [22].

## Data Availability

Data sharing is not applicable.

## Abbreviations

ALS	antiporter-like subunits
cryoEM	cryo-electron microscopy
dQ	decylubiquinone
EPR	electron paramagnetic resonance spectroscopy
FC	ferricyanide
FP	three subunit fragments solubilized from complex I upon chaotropic treatment
FSQ**^•−^** and FSQH^•^	deprotonated and protonated flavosemiquinone, respectively
HAR	hexaammineruthenium (III)
MD	membrane domain of complex I
PD	peripheral domain of complex I
*pmf*	proton motive force
RET	reverse electron transfer
ROS	reactive oxygen species
SBPs	subbacterial particles (plasma membrane vesicles)
SMPs	submitochondrial particles
TMH	transmembrane helix
Q	ubiquinone-10
